# General practitioners’ experiences of providing lifestyle advice to patients with depression: A qualitative focus group study

**DOI:** 10.1371/journal.pone.0299934

**Published:** 2024-03-11

**Authors:** Emma Astaire, Laura Jennings, Martina Khundakar, Sergio A. Silverio, Angela C. Flynn

**Affiliations:** 1 School of Life Course & Population Sciences, Faculty of Life Sciences & Medicine, King’s College London, London, United Kingdom; 2 Pharmacy Department, Cumbria, Northumberland, Tyne and Wear NHS Foundation Trust, Newcastle-upon-Tyne, United Kingdom; 3 School of Pharmacy, Newcastle University, Newcastle-upon-Tyne, United Kingdom; 4 School of Psychology, Faculty of Health, Liverpool John Moores University, Liverpool, United Kingdom; 5 School of Population Health, Royal College of Surgeons in Ireland, Dublin, Ireland; Keele University, UNITED KINGDOM

## Abstract

**Objective:**

Depression is an increasingly common mental health disorder in the UK, managed predominantly in the community by GPs. Emerging evidence suggests lifestyle medicine is a key component in the management of depression. We aimed to explore GPs’ experiences, attitudes, and challenges to providing lifestyle advice to patients with depression.

**Method:**

Focus groups were conducted virtually with UK GPs (May-July 2022). A topic guide facilitated the discussion and included questions on experiences, current practices, competence, challenges, and service provision. Data were analysed using template analysis.

**Results:**

‘Supporting Effective Conversations’; ‘Willing, but Blocked from Establishing Relational Care’; ‘Working Towards Patient Empowerment’; and ‘Control Over the Prognosis’ were all elements of how individualised lifestyle advice was key to the management of depression. Establishing a doctor-patient relationship by building trust and rapport was fundamental to having effective conversations about lifestyle behaviours. Empowering patients to make positive lifestyle changes required tailoring advice using a patient-centred approach. Confidence varied across participants, depending on education, experience, type of patient, and severity of depression.

**Conclusions:**

GPs play an important role in managing depression using lifestyle medicine and a patient-centred approach. Organisational and educational changes are necessary to facilitate GPs in providing optimal care to patients with depression.

## Introduction

Depression is a common psychiatric illness characterised by at least two weeks of low mood accompanied by a range of associated physical, emotional, behavioural, and cognitive symptoms [1]. Mental ill-health costs the National Health Service (NHS) approximately £119 billion in 2019/20 [2]. The increasing prevalence and economic burden of depression makes it a pressing public health issue. The National Institute for Health and Care Excellence (NICE) recommends antidepressants, talking therapy, and physical activity programmes, either separately or in combination to treat mild to moderate depression. In line with the growing evidence for lifestyle interventions to treat depression, NICE guidelines for less severe depression were recently updated and now include a recommendation to provide advice on maintaining a healthy lifestyle [3].

Mental illness, including depression ‐ the focus of this manuscript ‐ is associated with sub-optimal health behaviours such as poor diet, reduced physical activity, poor sleep, smoking and alcohol misuse [4–11]. There is an emerging evidence-base demonstrating that improved lifestyle behaviours can be important in the treatment of depression [12, 13]. Co-morbidity, including cardiovascular disease and diabetes, is high in patients with depression which increases the risk of morbidity and mortality [14–17].

General Practitioners (GPs), also known as ‘Family Doctors’ play a pivotal role in the management of depression with 90% of adults with mental ill-health being managed in primary care in the UK [18, 19]. GPs are expected to incorporate lifestyle interventions into depression management consultations. A key component of lifestyle interventions is supporting sustainable changes using behaviour change techniques such as motivational interviewing and patient-centred care [20, 21]. However, research suggests doctors lack lifestyle medicine education and knowledge [22]. Specifically, UK-based GPs lack adequate training on providing physical activity advice and recognise they have limited nutrition knowledge while medical students lack knowledge on diet in relation to chronic diseases [23, 24]. GPs may therefore be limited in their ability to incorporate lifestyle advice in the management of patients with depression.

The aim of this study was to explore UK GPs’ experiences, attitudes, and challenges to providing lifestyle advice to patients with depression.

## Methods

### Design & ethical approvals

Focus groups were considered to be the most comprehensive way of assessing a broad scope of opinion from multiple participants on this relatively exploratory topic, in a relatively short space of data collection period [25]. We therefore approached this study as philosophically pragmatic [26]–both in terms of ontology (whereby we acknowledge differing and sometimes competing interpretations of the world exist, and that no single viewpoint is able to provide the whole picture–thus scoping many opinions via a focus group was deemed best practice to capture these differing opinions) [27], and epistemology (whereby we accept that the knowledge and reality held and lived by people, is measurable in the real world accounting for time and cultural shift) [28]. Ethical approvals were obtained from the King’s College London Research Ethics Committee (ref:- LRU/DP-21/22-28890). Verbal, audio recorded consent was also taken before the commencement of each focus group to ensure participants were happy to participate in the study. Participants were made aware of their right to withdraw and were fully debriefed after focus groups.

### Participants

We recruited GPs (N = 16; five male; eleven female) across four focus group discussions. Participants were recruited using an advertisement template via email chains and social media platforms. Those who participated were UK-based practising GPs, with varied amounts of years of experience working in primary care (*M*_*Years*_ = 10; *Range* = 2–22 years). Participants worked in a variety of UK-based locations including Scotland, London, West and East England, and the Midlands. Two participants were excluded from the data analysis due to technical issues during the focus group and therefore 14 GPs (four male; ten female) were included in the final analysis.

### Data collection

The focus groups were conducted via Microsoft Teams and took place between May and July 2022. The interviews lasted 46-67minutes (*M*_*Time*_ = 60minutes), and were moderated by two Master’s students (a Junior Doctor [EA], and a Psychology graduate [LJ]). Participants provided informed consent, before the questioning began. A common set of questions were used as a topic guide (S1 Appendix) for each focus group, however, pertinent conversations raised by each individual group were followed-up on by the moderators. Data were recorded and the audio transcribed ‘intelligently’ (i.e., without non-verbal utterances), ready for analysis.

### Approach to analysis

A Template Analysis was selected to analyse the data, which is methodically stepped first refamiliarization with the data; then preliminary coding; organising themes; defining the initial coding template; applying the initial template; before finalising the template and applying it to the full dataset [29, 30]. The first pass analyses were completed independently by two authors (EA, LJ). Importantly, the initial template can be modified to ensure completeness of analysis, which was undertaken by another author (SAS), to harmonise any discordant themes as presented in the initial analyses [29]. Regular analytic discussion took place between all authors to ensure rigour, and thematic concordance.

## Results

Analysis identified four key themes: 1) Supporting Effective Conversations; 2) Willing, but Blocked from Establishing Relational Care; 3) Working Towards Patient Empowerment; and 4) Control Over the Prognosis. Each theme is presented below with the most eloquent quotations (additional supporting quotations can be found in *Table 1*).

**Table 1:** Additional supporting quotations.

Theme 1	Theme 2	Theme 3	Theme 4
“I think because I feel that patients are ready for it, quite a lot of them, maybe not during the first consultation but when you know that you’ve spoken to them before and they’ve gained your trust maybe a little bit, and if you’re working in the same surgery you know your patients, it’s a little bit easier to then embark on that.” (GP1)	“Time is a huge barrier to that and I often rush over it really quickly and it’s probably not very helpful advice, because it’s so general because I haven’t got time to be more specific.” (GP12)	“I try and build lifestyle advice into the assessment …very open and vague openers to find out what they’re actually doing at the moment …I think it’s really important to ask them questions because that has implications for their understanding of its relevance …I take it from there really, so I’m kind of led by them”. (GP8)	“Social Prescribers are great for lots of situations, but I do find that I probably refer a proportionally higher amount of elderly people who are struggling with loneliness and mental health and have found that they’re incredibly helpful in terms of giving them more lifestyle advice.” (GP12)
	“Probably having the least experience … I don’t think you are very well equipped. And other than looking at the basic resources that patients can look at, you might not know how to tailor something. So I think confidence is one thing and I think when you’ve been doing GP for a long time, certainly five years post, you become a lot more experienced just generally.” (GP3)		
			“For my nutrition, I say they need to watch a video from Tim Spector or from ZOE … that’s where the research is. And it’s quite accessible.” (GP9)
		“I think just asking about their daily routine gives you a lot of those answers so you don’t actually have to specifically say let’s talk about nutrition, or let’s talk about exercise …I feel it’s not as threatening a question” (GP5)	
“It might take me three or four appointments with someone before, “I know what you do now, I can visualise you at home, I know what your daily setup is, I know the barriers.” … I’d say the key thing is spending that bit of extra time to get to know someone, trying to give them that continuity.” (GP3)			
			“Unfortunately sometimes for patients they are cost prohibitive, and I think if there was something like a Yoga group run on the NHS, I think that would actually be great …” (GP1)
	“I think that knowledge around the lifestyle advice is not something that we went into much depth in at medical school so it’s something you sort of pick up.” (GP11)		
		““They took two groups of patients and one of them was given Psychology treatment or counselling and the other group was counselled in detail how to improve their diet. And guess what? The diet group was performing better.” So, I think these kinds of examples do help to make patients listen and maybe start slowly changing their dietary patterns.” (GP1)	
			“And there are outdoor gyms which is something that I do direct people to but other than that, everything comes with a price tag really unless you can start with a small trial at a council gym, but even those are pretty hard to come by …” (GP13).
“The nature of being teenagers they will be reluctant, but I think if you can just get them on board on your side, if you can make them feel that you’re not judging them, that you’re going to work with them, you’re there just to listen to them, you’re their advocate, and you get them on board, I think you can really make a big difference.” (GP4)			
	“I think since I’ve qualified with a BSLM [British Society of Lifestyle Medicine] diploma, I have been a lot more confident in advising patients how else they can improve their health other than the usual prescriptions for antidepressants or referring them for further secondary care.” (GP1)		
			“We don’t have very good access to community dieticians … so we don’t really know what their approach is …I think you do need to know that you’re aligned with these people you’re referring to” (GP8)
		“I tend to ask them what would be the easiest thing to fit, and then let them think about it for a bit.” (GP8)	
	“I think as I’ve got more experienced, I’ve hopefully learnt a bit about trying to individualise what people might be able to do and try and nail down a little bit some more individualised targets and coaching type stuff, so that people can go out of a consultation with an idea of what they might do rather than generalising.” (GP13)	“The way I approach it with teenagers is asking about their hobbies and do they get out, do they like going outside? … just talking to them about whether they’re interested in any activities, and going from there.” (GP8)	
			“They’re a very sensitive group, and a lot of it then depends on their background; their family and their parents …” (GP10).
“I feel when it’s more like a chat, more like an informal conversation, you get to have a lot more key information …I make it very open, and I show interest in the situation …” (GP10)			
			“The parent has a massive impact so even if the child wants to eat really well, if the parents themselves struggle with that then …” (GP9)
“It’s actually a very sensitive thing patients’ lifestyle, so I think those micro-comments, the way you want to ask those questions, the way you say them …are very key and you have to be very sensitive about them”. (GP10)			
	“I feel confident in giving lifestyle advice to people with depression, and I train as a Health Coach as well so I’m having separate training for that, which is helpful. … I’m paying for it separately.” (GP8)	“Each time, along with his prescription for sertraline, I’d give him a prescription for something to do … Actually, one of the tasks was to just go and sit outside the house every day for ten minutes, or even if he can’t manage ten minutes, just two minutes.” (GP6)	“We cater for a lot of refugees and immigrant population, and they mostly have this vision that a doctor, they have a magic wand or something, and they expect “I will be given medication,” or “You will sort me out.” So, that becomes a barrier.” (GP5)
		“…she’d stopped doing all the things that she really liked like reading and going out, so just suggesting go two minutes in the garden, make sure you get sun on your face …I get a lot of patients saying the same thing like gosh I didn’t know such simple things could help and I’ve never felt so heard …” (GP3).	
			“…it can be trickier with different cultures so if you’re not as familiar with… . different diets, then that can make it harder to give lifestyle advice as well” (GP12).
			“We have a fitness club through the primary care network and so that’s where they’ll get free support once a week on walking and running and cycling and swimming.” (GP7)
		“If you’re not sleeping well your mood’s going to be affected, you are going to feel a bit more low mood…And then when you tie in the link with other aspects of their lifestyle, their exercise or whatever, they get it and they often appreciate the effort and the time that you’ve taken and made to explain it to them.” (GP4)	
			“What a patient might think is healthy for them can be very different from what is known to be healthy…some patients may feel that stopping smoking would be damaging to their body.” (GP8)
		“I actually tell a story of a patient who has very bad bipolar and she was just eating crap…and I just suggested once that she maybe start cooking her own food … she got significantly better… it was a happy ending, so it’s nice to tell people stories about other patients ‐ they don’t want to know about studies particularly.” (GP9)	
			“Quite often they want to do better with… their diet habits, but either they don’t have the time or the skills to cook, or if it’s an 18-year-old girl I saw recently, she said “Nobody cooks in my family, my mum doesn’t know how to cook so we just get takeaways or we go to McDonald’s.” (GP1)
		“Some could be depressed from the stress of having to take care of kids, so that gives me an idea of how to deal with the specific cases of patients. For some, I actually try to understand what they want to achieve … and that helps me to be able to do some kind of assessment and then help them with lifestyle advice.” (GP10)	

### Theme 1: Supporting effective conversations

In consultations for the management of depression, initial patient engagement and the timing of introducing lifestyle advice to patients was spoken about as being an important part of the process. Several participants expressed that the conversation was more effective over several appointments as readiness to engage can take time, requires building of trust and is important for the provision of tailored advice.

*“Often it’s that engagement that you have to do before you even broach lifestyle advice because if they’re not engaged with you*, *if they don’t feel that you’re listening*, *then they may get the wrong assumption*, *maybe thinking that you somehow think it’s their fault or that they can solve it*, *and that’s very much not what’s happening*.*”* (GP7)

Empathy, lack of judgement and building trust were highlighted as being important in conversations about lifestyle particularly for certain patient groups such as adolescents.

*“I think it’s more about you not appearing judgmental*, *not appearing like you’re trying to convince them of something*. *I think you should appear more like trying to understand them and their habits… and then try to see how you can motivate them*.*”* (GP10)

Some participants spoke about using personal experiences and anecdotes to build trust with their patients.

*“…just your own personal experiences and anecdotes* … *we still all have the same experiences as our patients* … *and I think if patients can relate to you as a person*, *as a human being* … *it’s these little things that can instill confidence in your patient* …*” (GP5)*.

### Theme 2: Willing, but blocked from establishing relational care

The participants showed willingness to discuss lifestyle advice with patients with depression but several challenges to establishing relational care were highlighted. Lack of time and not having in-person consultations were highlighted as impacting practice. Telephone consultations were viewed as challenging as opportunities to pick-up on non-verbal cues are missed.

*“I think it’s sometimes lack of time and trying to squeeze it all in a ten-minute consultation*, *and most of the stuff is now done over the phone… you’re also missing out on the body language*, *on other non-verbal cues*.*”* (GP1)

The participants varied in confidence in providing lifestyle advice. Experience was related to confidence and more experience helped participants develop the skills required to personalise lifestyle advice.

*“I would say I’m more confident on the mild-to-moderate ones*. *Definitely for the severe ones I’m not sure it’s particularly my confidence in it but just more knowing that there’s a lot more complexity going on that will need to be taken into account*.*”* (GP7)

The participants highlighted that doctors are not provided with adequate educational opportunities on lifestyle medicine during their training and often this resulted in not feeling qualified. Including more lifestyle medicine in educational curricula was suggested.

*“*… *I’m not necessarily confident in all of the content* … *I don’t feel like I’ve got enough of an evidence-based knowledge bank* … *it would be really useful if there was more preventative medicine and more lifestyle education as part of the undergraduate course* … *we’re not as skilled in it as we should be really* …*” (GP12)*

### Theme 3: Working towards patient empowerment

The participants spoke about introducing the topic of lifestyle by asking open questions about daily routines and then using the information gained to personalise the discussion.

*“I say something like ‘Talk me through your day’*, *so I can understand where they are at the moment …*.*and see whether they think there’s anything that they could fit into what they do at the moment*.*”* (GP9)

Participants spoke about taking a patient-centred approach and using shared decision making to empower the patient to take control of the management of their health.

*“They need their own way to feel empowered and their own thing that’s going to pull them forward is digging into what was important to them*, *so that’s what I tend to try to focus on*.*”* (GP3)

Understanding the patients’ interests and goals was discussed by the participants as being an important component of counselling. This was spoken about in the context of postpartum women and adolescent patients. Introducing realistic, specific and achievable changes was highlighted by the participants as an important part of lifestyle medicine. Often, the participants would focus on one or two goals at a time.

*“‘Can you go outside*?*’ that could be like climbing Everest for that patient… Opening the curtains*, *that was one of the goals that we set with one of my patients… I think it’s shifting those goalposts*.*”* (GP3)

### Theme 4: Control over the prognosis

The importance of access to additional support, such as social prescribers, specialist mental health nurses, or other mental health practitioners was discussed by the participants. These specialists were viewed as having more up-to-date knowledge and better awareness of what resources are available to patients and hence can contribute to better care, and despite being based in the community, were linked to specialist mental health Trusts.

*“If we do have a Mental Health Nurse*, *she is much more up to date with what else is going on and what else can we do*.*”* (GP1)

Access to resources which are often free to support patients with lifestyle changes was discussed. Participants spoke about different resources including local physical activity clubs, podcasts, and websites. However, the participants highlighted how economic challenges impact patients’ ability to make lifestyle changes, such as access to exercise classes, dietitians, and resources which are not routinely available on the NHS.

*“I think about my local area*, *we kind of have most of the things you would want*, *it’s just that they might be under-resourced or underfunded or oversubscribed or difficult to access*.*”* (GP12)

Conversations with particular groups of patients were spoken about as being associated with additional considerations. For example, the participants acknowledged the importance of discussing lifestyle changes with patients with postpartum depression. However, some aspects such as sleep were highlighted as untimely to discuss in the postpartum period.

*“As much as you’d like to talk about lifestyle medicine… it’s just not going to work for them because they’re not going to sleep; it’s a major issue and that’s just a part and parcel of being a new mother… I think trying to go down the lifestyle medicine route might come across as being a bit condescending to them*.*”* (GP4)

Likewise, the social environment was also perceived as a challenge for other groups such as parents.

*“People aren’t always entirely in charge of what they’re doing in terms of their lifestyle*. *Some people might want to go outside and exercise and they just don’t have that autonomy to do it*, *or they’re looking after their kids*, *and it’s just finding out what exactly is the barrier there and seeing if there’s a way around it*.*”* (GP8)

## Discussion

### Summary of main findings

Establishing a doctor-patient relationship by building trust and rapport was fundamental in having effective conversations on making lifestyle changes as an adjunct to the management of depression. Additionally, empowering patients to make positive lifestyle changes requires tailoring advice using a patient-centred approach. Confidence in lifestyle advice provision varied between the participants, and was associated with differing training, education, experience, severity of depression and patient sub-group. A common organisational challenge to establishing relational care was lack of time. It is well-established that lack of time is a barrier to lifestyle advice provision in chronic disease management in primary care [31–34]. This is despite in 2014 the Royal College of GPs proposing that GPs should be allotted longer consultations for patients with mental ill-health [35]. The participants further highlighted longer consultations are required for patients with depression due to the individualised approach required when giving lifestyle advice and due to the complexity of patients with mental ill-health. Therefore, increasing appointment length could have a significant positive impact on the care of patients with depression and facilitate conversations on lifestyle changes. Additionally, participants highlighted telephone consultations are disruptive to establishing relational care as non-verbal cues are missed. Although there is evidence suggesting telemedicine can be beneficial to patients with depression due to increased access to care [36], emphasis on in-person consultations in primary care for patients with mental ill-health is still needed to ensure patients with depression receive individualised advice on behaviour change.

The participants found patient-led decision-making was effective for empowering patients to make lifestyle changes, with the patient directing the doctor on which area of lifestyle to focus on. Further evidence suggests that a patient-centred approach appears to be valued by patients with depression and may be associated with better treatment outcomes [37, 38]. Tailoring the lifestyle advice for patient subgroups, such as adolescents, postpartum women, and those with severe depression was also evident. Another key component to patient empowerment was educating the patient about the link between lifestyle and mood. Some participants provided evidence-based lifestyle advice from existing research, while others used patient anecdotes [39]. Empowering patients to make lifestyle changes using a patient-centred approach may lead to more effective management of depression using lifestyle medicine.

GPs identified economic and access challenges which may impact patients’ abilities to make lifestyle changes as supportive resources were location dependent, and some were costly. To achieve standardised care provision and reduce health service inequalities, the access to supportive and free resources needs to be standardised across NHS practices. This is particularly important as depression is more common in lower socio-economic groups [40]. Some of the GPs had access to social prescribing and mental health nurses and felt they were particularly supportive to patients with depression and were experts in the area. Evidence shows social prescribing can reduce levels of depression and improve quality of life [41]. Access to social prescribing should be addressed within primary care to improve healthcare outcomes for patients with depression.

### Strengths and limitations

This study provides knowledge to the limited body of qualitative research exploring GPs’ experiences of providing lifestyle advice to patients with depression. The participants who opted to participate in the study may be motivated to provide lifestyle advice which might have resulted in selection bias. Further, during the focus group interviews, two participants were lost from the focus groups due to technical issues and therefore also from the overall dataset, reducing the sample size of this study and the amount of data collected. The study unfortunately did not have a participant group which reflects the general UK GP workforce, which has a lower proportion of male to female participants. Finally, further information on the GP practices such as the practice size was not gathered and future work should be cognisant of these socio-demographic factors. The sampled participants did however provide strength to the data as the GPs were from both urban and rural areas across the UK. Having a varied representation of UK settings is useful in determining how location can influence provision of lifestyle advice and access to resources. Additionally, the participants had a range of experience which is important given that experience was found to influence confidence.

## Conclusion

GPs can play an important role in managing depression using lifestyle medicine. Together, the current primary care structure and educational provision needs review to ensure GPs can provide optimal care to patients with depression in line with national guidance.

## Supporting information

S1 Appendix (PDF)

## Abbreviations

UK: United Kingdom
NICE: National Institute for Health and Care Excellence
GP: General Practitioner

## References

[1] American Psychiatric Association. Diagnostic and Statistical Manual of Mental Disorders: Diagnostic and Statistical Manual of Mental Disorders, Fifth Edition. American Psychiatric Association; 2013.

[2] O’Shea N, Bell A. A Spending Review for Wellbeing.; 2020.

[3] National Institute for Health and Care Excellence (NICE). Depression in adults: treatment and management NICE guideline [NG222]. Published June 29, 2022. Accessed July 21, 2022. https://www.nice.org.uk/guidance/ng222/chapter/Recommendations#treatment-for-a-new-episode-of-less-severe-depression%20%20https://www.nice.org.uk/guidance/cg90/evidence/full-guideline-pdf-4840934509. 35977056

[4] Firth J, Solmi M, Wootton RE, et al. A meta‐review of “lifestyle psychiatry”: the role of exercise, smoking, diet and sleep in the prevention and treatment of mental disorders. World Psychiatry. 2020; 19(3): 360–380. doi: 10.1002/wps.20773 32931092 PMC7491615

[5] Jacka FN, Pasco JA, Mykletun A, et al. Association of Western and traditional diets with depression and anxiety in women. American journal of psychiatry. 2010;167(3):305–311. doi: 10.1176/appi.ajp.2009.09060881 20048020

[6] An R, Xiang X. Smoking, heavy drinking, and depression among US middle-aged and older adults. Prev Med (Baltim). 2015;81:295–302. 10.1016/j.ypmed.2015.09.026 26436684

[7] Jerstad SJ, Boutelle KN, Ness KK, Stice E. Prospective reciprocal relations between physical activity and depression in female adolescents. J Consult Clin Psychol. 2010;78(2):268. doi: 10.1037/a0018793 20350037 PMC2847789

[8] Hawkley LC, Capitanio JP. Perceived social isolation, evolutionary fitness and health outcomes: a lifespan approach. Philosophical Transactions of the Royal Society B: Biological Sciences. 2015;370(1669):20140114. doi: 10.1098/rstb.2014.0114 25870400 PMC4410380

[9] Jacka FN, Cherbuin N, Anstey KJ, Butterworth P. Does reverse causality explain the relationship between diet and depression? J Affect Disord. 2015;175:248–250. doi: 10.1016/j.jad.2015.01.007 25658499

[10] Ruusunen A, Lehto SM, Mursu J, et al. Dietary patterns are associated with the prevalence of elevated depressive symptoms and the risk of getting a hospital discharge diagnosis of depression in middle-aged or older Finnish men. J Affect Disord. 2014; 159:1–6. doi: 10.1016/j.jad.2014.01.020 24679382

[11] Jacka FN, Mykletun A, Berk M et al. The association between habitual diet quality and the common mental disorders in community-dwelling adults: the Hordaland Health study. Psychosom Med. 2011; 73(6):483–90. doi: 10.1097/PSY.0b013e318222831a 21715296

[12] Sarris J, Kavanagh DJ, Newton R. Depression and exercise. Journal of Complementary Medicine: CM, The. 2008;7(3).

[13] Sarris J, Logan AC, Akbaraly TN, et al. Nutritional medicine as mainstream in psychiatry. Lancet Psychiatry. 2015;2(3):271–274. doi: 10.1016/S2215-0366(14)00051-0 26359904

[14] O’Neil A, Jacka FN, Quirk SE, et al. A shared framework for the common mental disorders and non-communicable disease: key considerations for disease prevention and control. BMC Psychiatry. 2015;15(1):1–6. doi: 10.1186/s12888-015-0394-0 25652365 PMC4342822

[15] Moussavi S, Chatterji S, Verdes E, Tandon A, Patel V, Ustun B. Depression, chronic diseases, and decrements in health: results from the World Health Surveys. The Lancet. 2007;370(9590):851–858. doi: 10.1016/S0140-6736(07)61415-9 17826170

[16] Chesney E, Goodwin GM, Fazel S. Risks of all‐cause and suicide mortality in mental disorders: a meta‐review. World psychiatry. 2014;13(2):153–160. doi: 10.1002/wps.20128 24890068 PMC4102288

[17] Public Health England. Severe mental illness (SMI) and physical health inequalities: briefing. Published September 27, 2018. Accessed July 21, 2022. https://www.gov.uk/government/publications/severe-mental-illness-smi-physical-health-inequalities/severe-mental-illness-and-physical-health-inequalities-briefing.

[18] The Mental Health Taskforce. The Five Year Forward View for Mental Health.; 2016.

[19] British Medical Association. Breaking down Barriers–the Challenge of Improving Mental Health Outcomes.; 2017.

[20] Egger G. Lifestyle medicine:’The’why’,’what’and’how’of a developing discipline’. Aust J Gen Pract. 2019;48(10):665–668. 10.31128/AJGP-06-19-4955 31569327

[21] Araújo-Soares V, Hankonen N, Presseau J, Rodrigues A, Sniehotta FF. Developing behavior change interventions for self-management in chronic illness: An integrative overview. Eur Psychol. 2019;24(1):7. doi: 10.1027/1016-9040/a000330 31496632 PMC6727632

[22] Crowley J, Ball L, Hiddink GJ. Nutrition in medical education: a systematic review. Lancet Planet Health. 2019;3(9):e379–e389. doi: 10.1016/S2542-5196(19)30171-8 31538623

[23] Chatterjee R, Chapman T, Brannan MGT, Varney J. GPs’ knowledge, use, and confidence in national physical activity and health guidelines and tools: a questionnaire-based survey of general practice in England. British Journal of General Practice. 2017;67(663):e668–e675. doi: 10.3399/bjgp17X692513 28808077 PMC5604830

[24] Macaninch E, Buckner L, Amin P, et al. Time for nutrition in medical education. BMJ Nutr Prev Health. 2020;3(1):40. doi: 10.1136/bmjnph-2019-000049 33235970 PMC7664491

[25] Luke M, Goodrich KM. Focus Group Research: An Intentional Strategy for Applied Group Research? The Journal for Specialists in Group Work. 2019;44(2):77–81. doi: 10.1080/01933922.2019.1603741

[26] Morgan D. L. (2014). Pragmatism as a paradigm for social research. Qualitative inquiry, 20(8), 1045–1053.

[27] Woodring J. C., Foley S. M., Santoro Rado G., Brown K. R., & Hamner D. M. (2006). Focus groups and methodological reflections: Conscientious flexibility in the field. Journal of Disability Policy Studies, 16(4), 248–258.

[28] Ruwhiu D., & Cone M. (2010). Advancing a pragmatist epistemology in organisational research. Qualitative Research in Organizations and Management: An International Journal, 5(2), 108–126.

[29] King N. Doing Template Analysis. Qualitative Organizational Research: Core Methods and Current Challenges. Vol 426. SAGE Publications Ltd; 2012.

[30] Brooks J, McCluskey S, Turley E, King N. The Utility of Template Analysis in Qualitative Psychology Research. Qual Res Psychol. 2015;12(2):202–222. doi: 10.1080/14780887.2014.955224 27499705 PMC4960514

[31] Douglas F, Torrance N, van Teijlingen E, Meloni S, Kerr A. Primary care staff’s views and experiences related to routinely advising patients about physical activity. A questionnaire survey. BMC Public Health. 2006;6(1):1–10. doi: 10.1186/1471-2458-6-138 16719900 PMC1523207

[32] Lambe B, Collins C. A qualitative study of lifestyle counselling in general practice in Ireland. Fam Pract. 2010;27(2):219–223. doi: 10.1093/fampra/cmp086 20008029

[33] Geense WW, van de Glind IM, Visscher TLS, van Achterberg T. Barriers, facilitators and attitudes influencing health promotion activities in general practice: an explorative pilot study. BMC Fam Pract. 2013;14(1):1–10. doi: 10.1186/1471-2296-14-20 23394162 PMC3575260

[34] Crowley J, Ball L, McGill AT, et al. General practitioners’ views on providing nutrition care to patients with chronic disease: a focus group study. J Prim Health Care. 2016;8(4):357–364. doi: 10.1071/HC15048 29530161

[35] British Medical Association. Breaking down Barriers–the Challenge of Improving Mental Health Outcomes.; 2017.

[36] Felix de Oliveira PB, Dornelles TM, Gosmann NP, Camozzato A. Efficacy of telemedicine interventions for depression and anxiety in older people: A systematic review and meta-analysis. Int J Geriatr Psychiatry. 2023; 38(5):e5920. doi: 10.1002/gps.5920 37204341

[37] Grung I, Anderssen N, Haukenes I et al. Patient experiences with depression care in general practice: a qualitative questionnaire study. Scand J Prim Health Care. 2022; 40(2):253–260. doi: 10.1080/02813432.2022.2074069 35603990 PMC9397414

[38] Jani B, Bikker AP, Higgins M et al. Patient centredness and the outcome of primary care consultations with patients with depression in areas of high and low socioeconomic deprivation. Br J Gen Pract. 2012; 62(601):e576–81. doi: 10.3399/bjgp12X653633 22867682 PMC3404336

[39] Jacka FN, O’Neil A, Opie R, et al. A randomised controlled trial of dietary improvement for adults with major depression (the ‘SMILES’trial). BMC Med. 2017;15(1):1–13. 28137247 10.1186/s12916-017-0791-y PMC5282719

[40] World Health Organization. Social Determinants of Mental Health.; 2014.

[41] Chatterjee HJ, Camic PM, Lockyer B, Thomson LJM. Non-clinical community interventions: a systematised review of social prescribing schemes. Arts Health. 2018;10(2):97–123.

