# Supplementary material for: General practitioners’ experiences of providing lifestyle advice to patients with depression: A qualitative focus group study
Source: PLoS One. 2024 Mar 11;19(3):e0299934. doi: 10.1371/journal.pone.0299934 (PMC10927082; doi:10.1371/journal.pone.0299934)
Supplement: S1 Appendix — (PDF) [file pone.0299934.s001.pdf]

Topic Guide for General Practitioners – 'An exploration of general practitioner's views and experiences of providing lifestyle advice to individuals with mental illness'

Intro

We are two nutrition MSc students conducting a research project to determine General Practitioners' views, experiences, attitudes and barriers towards giving lifestyle advice to patients with depression within primary care.

In this hour focus group discussion we will cover current practice, competence, barriers and service provisions in regards to lifestyle advice with a focus on depression.

Lifestyle Advice – focus on mild to moderate depression

1) *Current practice*

- In your own practice, what are your experiences of giving lifestyle advice to patients with depression?
    - If not mentioned
        - what about physical activity?
        - What about nutrition?
        - What about sleep / sociability?

- And have you had any similar experiences of giving the above advice to
    - Women with post-partum depression
    - People with severe depression
    - adolescents / children with depression
    - Elderly persons with depression

Competence, Confidence & Service Provision in providing lifestyle advice to patients with depression

- How confident are you in providing lifestyle advice to any of the above patient groups with depression?

- Why do you feel confident or not confident in providing lifestyle advice to any of the previously discussed patient groups with depression?

- What helps or facilitates you in giving lifestyle advice?
    - If not covered ask about local services for social prescribing, weight management, dietician, smoking cessation, additional services.
    - If not mentioned probe about barriers

- What feedback have you received from patients with depression on these services?
- If no mention of relevant services ask what provisions could be useful?

Specialist Role

GPs can undertake an additional role within their core general practice that is beyond the scope of GP training and the MRCGP and requires further training otherwise known as an 'extended GP role'. The extended role is typically undertaken within a contract or setting that distinguishes it from a standard general practice. There is currently not an extended GP role that focusses on mental health.

- What are your thoughts on an Extended Role in Mental Health?
- How might this effect the care and prognoses for any of the patient groups with depression we have previously discussed?
